# Whole genome resequencing reveals signatures of rapid selection in a virus‐affected commercial fishery

**DOI:** 10.1111/mec.16499

**Published:** 2022-05-31

**Authors:** Owen J. Holland, Madeline Toomey, Collin Ahrens, Ary A. Hoffmann, Laurence J. Croft, Craig D. H. Sherman, Adam D. Miller

**Affiliations:** ^1^ 2104 School of Life and Environmental Sciences Deakin University Warrnambool Victoria Australia; ^2^ 2104 Deakin Genomics Centre Deakin University Geelong Victoria Australia; ^3^ 7800 School of Biotechnology and Biomolecular Sciences University of New South Wales Sydney Australia; ^4^ Research Centre for Ecosystem Resilience Australian Institute of Botanical Science Royal Botanic Garden Sydney New South Wales Australia; ^5^ School of BioSciences Bio21 Institute The University of Melbourne Parkville Victoria Australia

**Keywords:** blacklip abalone, genetic adaptation, haliotid herpesvirus‐1, infectious diseases, southeastern Australia, whole genome resequencing

## Abstract

Infectious diseases are recognized as one of the greatest global threats to biodiversity and ecosystem functioning. Consequently, there is a growing urgency to understand the speed at which adaptive phenotypes can evolve and spread in natural populations to inform future management. Here we provide evidence of rapid genomic changes in wild Australian blacklip abalone (*Haliotis rubra*) following a major population crash associated with an infectious disease. Genome scans on *H*. *rubra* were performed using pooled whole genome resequencing data from commercial fishing stocks varying in historical exposure to haliotid herpesvirus‐1 (HaHV‐1). Approximately 25,000 single nucleotide polymorphism loci associated with virus exposure were identified, many of which mapped to genes known to contribute to HaHV‐1 immunity in the New Zealand pāua (*Haliotis iris*) and herpesvirus response pathways in haliotids and other animal systems. These findings indicate genetic changes across a single generation in *H*. *rubra* fishing stocks decimated by HaHV‐1, with stock recovery potentially determined by rapid evolutionary changes leading to virus resistance. This is a novel example of apparently rapid adaptation in natural populations of a nonmodel marine organism, highlighting the pace at which selection can potentially act to counter disease in wildlife communities.

## 1 INTRODUCTION

The spread of infectious diseases is recognized as one of the most pressing global threats to biodiversity and ecosystem function (Cunningham et al., 2017; Daszak et al., 2000; Tompkins et al., 2015). In recent decades, infectious diseases have devastated a range of wildlife groups (Berger et al., 1998; Hansen et al., 2005; Kim & Harvell, 2004; Lorch et al., 2016), often exacerbating species declines in ecosystems already stressed by climate change and habitat destruction (Bosch et al., 2018; Brearley et al., 2013; Harvell et al., 2002). The persistence of many species will probably depend on their ability to adapt to environmental changes associated with increased disease prevalence, although selection for disease resistance or tolerance may not keep pace with rates of pathogen evolution and the emergence and turnover of novel diseases (Hawley et al., 2013; Ujvari et al., 2014).

Detecting rapid evolutionary changes in populations impacted by environmental disturbance is often challenging. Disease‐affected populations provide ideal models given disease exposure is an easily characterized selective pressure and studies of this nature have been greatly assisted by modern genomic technologies (Blanchong et al., 2016; Storfer et al., 2020). These technologies now allow for rapid and cost‐effective estimates of genome‐wide variation among populations spanning disease infection gradients and individuals with distinctive phenotypes related to disease response (Auteri & Knowles, 2020; Elbers et al., 2018; Grogan et al., 2018; Margres et al., 2018). Importantly, a number of studies using these technologies have reported rapid evolutionary changes across several generations in natural populations of nonmodel organisms impacted by disease, including Tasmanian devils (*Sarcophilus harrisii*) (Epstein et al., 2016; Hubert et al., 2018; Margres et al., 2018) and North American house finches (*Carpodacus mexicanus*) (Bonneaud et al., 2011). Additionally, recent studies have reported evidence of rapid selection for disease‐resistant genotypes across a single generation in North American sea stars (*Pisaster ochraceus*) and little brown bats (*Myotis lucifugus*), following rapid and severe population crashes due to infectious diseases (Auteri & Knowles, 2020; Schiebelhut et al., 2018). Such studies are pivotal in highlighting the pace at which selection can act to counter disease in wildlife communities and opening up opportunities for interventions, such as deliberate translocations of adaptive phenotypes, that can increase the adaptability of threatened populations (Hoffmann et al., 2020; Hohenlohe et al., 2019). Despite this progress, the number of studies demonstrating rapid evolutionary responses to infectious diseases in natural populations remains limited and biased towards terrestrial systems.

Marine infectious diseases are responsible for incremental and mass mortalities in a variety of wildlife groups, including keystone and habitat‐forming taxa (Clemente et al., 2014; Harvell & Lamb, 2020; Harvell et al., 2007; Martin et al., 2016; Montecino‐Latorre et al., 2016), and species supporting wild commercial fisheries (Cawthorn, 2011; Crosson et al., 2020; Lafferty et al., 2015; Marty et al., 2010). The Australian blacklip abalone (*Haliotis rubra*), a species targeted by the world’s largest wild abalone fisheries and a rapidly expanding aquaculture industry (FAO FishStat, 2021), was heavily impacted by disease between 2006 and 2010 (Mayfield et al., 2012). Beginning in 2006, abalone viral ganglioneuritis (AVG) caused by the haliotid herpesvirus‐1 (HaHV‐1) spread along the western coastline of Victoria in southeastern Australia, causing rapid and severe population collapses (>90% mortality in some areas) and devastating both wild and farmed abalone stocks (Hooper et al., 2007). Despite the impact of AVG, abalone stocks in the Western Zone fishery have seen significant recovery (Western Abalone Divers Association, 2020). It is possible that rapid evolutionary responses to the virus have contributed to this recovery, facilitated by the abalone’s short generation time (~4 years; Andrews, 1999), large population sizes (Mayfield et al., 2012) and high genetic variability that contributes to existing patterns of adaptation across the fishery (Miller et al., 2019).

Evolving immunity to HaHV‐1 depends on the availability of specific, heritable genetic variants within abalone populations that lead to resistant phenotypes. Indeed, previous research has demonstrated heritable genetic variation relating to herpesvirus immunity in sister Haliotid species, highlighting the possibility of evolved immunity in *H*. *rubra*. Challenge tests performed on New Zealand paua (*Haliotis iris*) and Japanese black abalone (*Haliotis discus*), involving controlled exposure to HaHV‐1, indicated complete immunity to AVG (Chang et al., 2005; Corbeil et al., 2017), with complementary transcriptomic analyses helping to characterize the genetic basis of the resistance (Bai, Zhang, et al., 2019; Neave et al., 2019). Similar tests on *H*. *rubra* yielded no evidence of resistance to AVG (Corbeil et al., 2016; Crane et al., 2013); however, these experiments were performed on animals from a limited number of locations affected by AVG. While complete immunity may not occur in *H*. *rubra*, the presence of AVG immunity in sister taxa hints at the potential for some level of resistance developing through standing genetic variation following AVG exposure.

For the first time in a decade AVG was recorded in 2021, leading to abalone mortalities at a few proximal fishing locations heavily impacted by AVG in the early 2000s (Agriculture Victoria, 2021). Unlike the first outbreak, animal mortality and disease spread has been minimal. While environmental and epidemiological factors may be contributing to the suppression of the disease (Bai, Li, et al., 2019; Corbeil, 2020), it is also possible that the mass mortality event from 2006–2010 selected for adaptive phenotypes which is reducing the number of susceptible animals and overall viral load within affected fishing stocks. To test this hypothesis, we investigate potential signatures of evolutionary changes in recovering *H*. *rubra* fishing stocks devastated by AVG. Specifically, we performed genome scans using pooled whole genome resequencing data on *H*. *rubra* specimens from fishing stocks varying in disease exposure. Our findings point to rapid changes in population‐level allele frequencies over a single generation timescale in virus‐affected fishing stocks, with stock recovery potentially determined by rapid evolutionary changes leading to virus resistance. This study highlights the pace at which adaptive phenotypes can potentially evolve and spread in wildlife communities to counter threats from infectious diseases. We discuss these findings in the context of future biosecurity management of Australian abalone fisheries and wildlife conservation more generally.

## 2 MATERIALS AND METHODS

### 2.1 Sample collection and DNA sequencing

**FIGURE 1:**
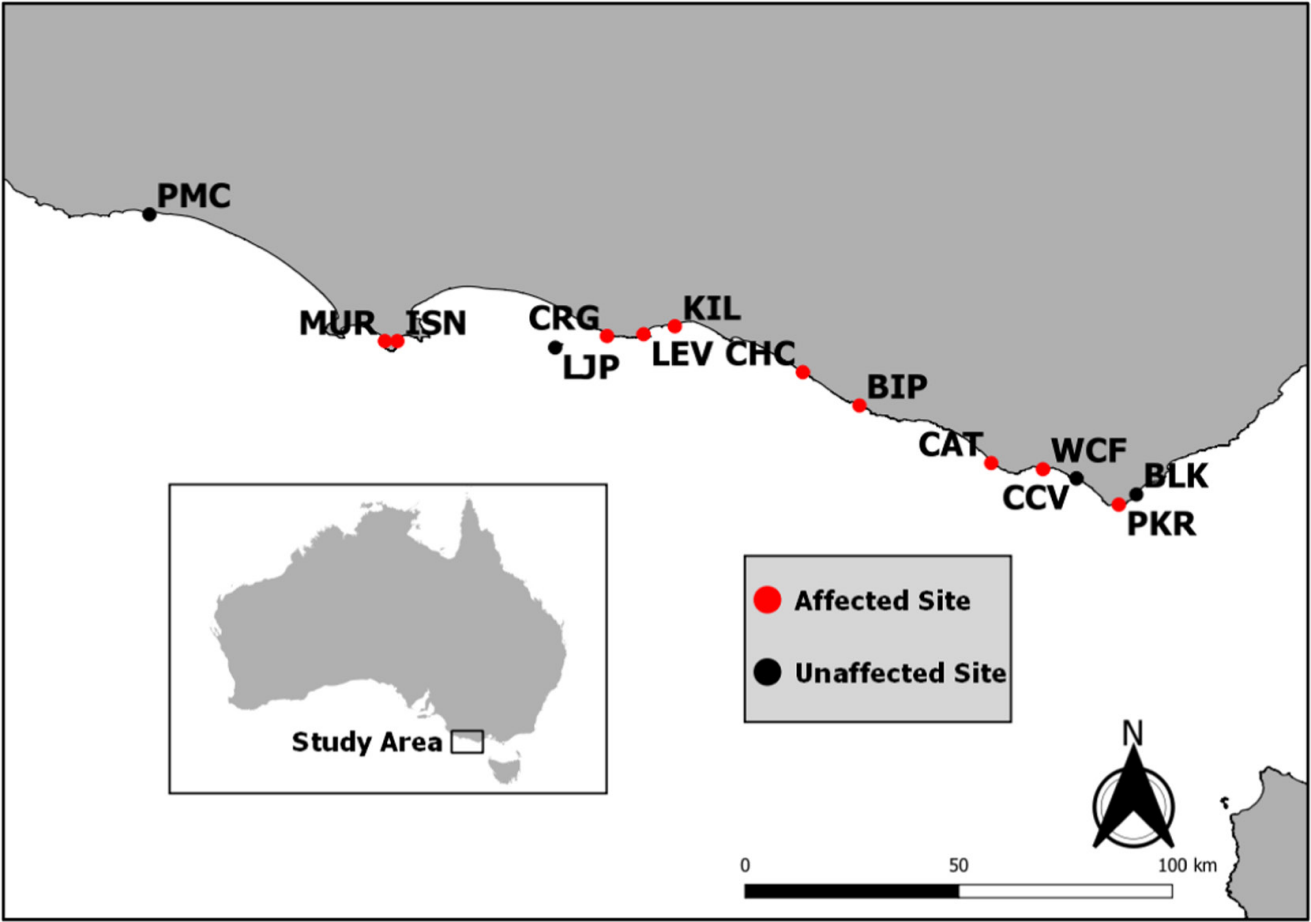
Sampling sites selected for population genomic analysis from southeastern Australia. Figure legend and colour coding of mapped sites indicate history of virus exposure. Refer to Table 1 for sample codes

Tissue biopsies were collected from 343 individual *Haliotis rubra* from 14 locations spanning the western and central coastline of Victoria. Locations were selected based on their known virus exposure history according to records held by the Victorian wild fishing sector and the Victorian Fisheries Authority. During the outbreak event, rigorous PCR (polymerase chain reaction) testing and diver surveys were conducted across the fishery to determine the spatial extent of the virus and rates of local mortality at affected sites, estimated from the abundance of dead and dying abalone and recently vacated shells (Gorfine et al., 2008). Based on these data we selected 10 AVG‐affected locations that showed >70% animal mortalities, and four AVG‐unaffected locations (Table 1, Figure 1). Sampling for five of the locations was coordinated in 2009 by the Department of Economic Development, Jobs, Transport and Resources (DEDJTR). It is expected that animals from these locations during this sampling period survived the virus event and represent the genomic variation preserved post‐AVG. Sampling of the remaining nine locations was performed between 2015 and 2020. To avoid the potential swamping effects of intergenerational gene flow since the disease outbreak, sampling was biased towards fishing stocks expected to be largely self‐recruiting based on biophysical connectivity models (Young et al., 2020), and toward large adult animals (expected to be either direct survivors or first‐generation post‐virus survivors). However, it is important to note that these fishing stocks are not completely self‐recruiting entities given previous studies have demonstrated a lack of genetic structure across the fishery (Miller et al., 2016, 2019). This sampling was performed by contract divers, commercial fishermen and our research team. At each location, individual abalone were collected within a 100‐m^2^ area, with tissue biopsies consisting of 20 mg of muscle tissue from the abalone lip obtained using sterile dissection tools to avoid cross‐contamination. Biopsied material was transferred to 2‐ml microcentrifuge tubes containing 80%–100% ethanol and stored at 4°C until required for genomic analysis.

**TABLE 1:** Site location details and corresponding codes for 14 collection locations of *Haliotis rubra* used for genomic analyses. Sample sizes and AVG exposure history are also provided

Zone and location	Code	Year sampled	Sample size	GPS location		AVG status
				Latitude	Longitude	
Port Macdonell	PMC	2020	25	–38.054	140.881	Unaffected
Inside Murrels	MUR	2009	25	–38.407	141.524	Affected
Inside Nelson	ISN	2009	25	–38.409	141.558	Affected
Lady Julia Percy	LJP	2009	23	–38.422	141.993	Unaffected
The Crags	CRG	2009	25	–38.390	142.135	Affected
Killarney	KIL	2015	20	–38.363	142.321	Affected
Levies	LEV	2009	25	–38.385	142.235	Affected
Childers Cove	CHC	2019	25	–38.490	142.672	Affected
Bay of Islands	BIP	2019	25	–38.582	142.827	Affected
Cat Reef	CAT	2015	25	–38.741	143.188	Affected
White Cliffs	WCF	2015	25	–38.758	143.330	Affected
Castle Cove	CCV	2020	25	–38.783	143.422	Unaffected
Parker River	PKR	2020	25	–38.855	143.538	Affected
Blanket Bay	BLK	2015	25	–38.827	143.586	Unaffected

Total genomic DNA was extracted from 10 mg of tissue using a DNeasy Blood and Tissue Kit (Qiagen) following the manufacturer’s instructions. Resulting DNA extracts were quantified using a Qubit version 2 fluorometer (Life Technologies). To obtain population genomic data, we applied the Pool‐Seq approach (Futschik & Schlotterer, 2010), which involves pooling the DNA of a large number of individuals from the same population and then sequencing the “population variability genome.” This was achieved by pooling equimolar amounts of individual DNA extracts from each sample location, splitting the 25 individuals per location into two pools per location consisting of DNA from 12 and 13 individuals, respectively, to account for potential sequencing bias. The resulting 28 pooled libraries were prepared for sequencing using the Nextera DNA Sample Preparation kit, pooled into a single Illumina NovaSeq S4 flowcell (Illumina) and sequenced across all four lanes with the 150‐bp paired‐end protocol. Sequencing was performed allowing for 3× genome coverage per individual per pool, equating to 80–100× genome coverage per population.

### 2.2 Data preparation

The Illumina NovaSeq sequencing yielded a total of 25 × 10^9^ assigned 150‐bp reads, and a total of 45–100 Gb of sequence data for each of the 28 pooled DNA libraries. Raw DNA sequence reads from the two separate pooled libraries per sample location were pooled for processing purposes. Raw sequences were processed using trimmomatic version 0.36 (Bolger et al., 2014) by removing Nextera adaptors and discarding all reads that had a Phred score below 20. All retained reads were subsequently aligned to the *H*. *rubra* reference genome (NCBI RefSeq QXJH00000000.1; Gan et al., 2019) using the ppalign package in the PoolParty pipeline (Micheletti & Narum, 2018) with default parameters. Single nucleotide polymorphisms (SNPs) were called using poolfstat (Hivert et al., 2018) where sites were required to have a read depth of 40–200 reads to be called. SNPs with a minor allele frequency of ≥0.05 were used for downstream genomic analysis.

### 2.3 | Estimating overall genetic structure

SNP frequencies over all loci were initially contrasted between all 14 sample locations to determine patterns of overall genetic structure and population connectivity. The software poolfstat implemented in R (Hivert et al., 2018) was used to calculate global and pairwise measures of population differentiation (*F*_ST_; Weir & Cockerham, 1984). Estimates of global and pairwise *F*_ST_ were also generated using a data set consisting of only candidate loci identified from association analyses.

### 2.4 | Genotype × environment association analysis

To identify SNPs associated with virus exposure status, we performed a genotype × environment association analyses using baypass 2.1 (Gautier, 2015). Analyses were performed under the auxiliary (AUX) covariate mode (‐covmcmc and ‐auxmode flags), after scaling the variables with the ‐scalecov flag, using input files containing genotypic data for each sampling location and corresponding virus exposure history (exposed vs. unexposed). The underlying models explicitly account for the covariance structure among the population allele frequencies that originates from the shared history of the populations through estimation of the population covariance matrix Ω, which removes the variation associated with demography (Bonhomme et al., 2010; Günther & Coop, 2013). The auxiliary covariate model specifically involves the introduction of a binary auxiliary variable to classify each locus as associated or not associated. This allows computation of posterior inclusion probabilities (and Bayes Factors) for each locus while explicitly accounting for multiple testing issues. The auxiliary covariate model was applied with default parameters, a 5000 burn‐in of iterations in the Markov chain Monte Carlo (MCMC) simulation, followed by 25,000 iterations. To reduce artefacts due to potential variability between runs, we performed three independent baypass simulations. We then calculated the average Bayes Factor (BF), expressed in deciban units (dB), for each SNP as a quantitative estimate of the strength of association with virus exposure and the standardized allele frequency. For each SNP, the level of effect was assessed based on the BF models according to Jeffrey’s rule (Jeffreys, 1961). SNPs with BF scores ≥50 were regarded as decisive associations with virus exposure and were retained as potential candidate loci.

### 2.5 Post hoc analyses including functional annotations

An analysis of principal components (PCA) was implemented in the adegenet package for R (Jombart, 2008; Jombart & Ahmed, 2011) to obtain a graphical depiction of patterns of genetic structure among virus‐affected and unaffected stocks based on all candidate SNPs identified by baypass (BFs ≥ 50).

Total linkage disequilibrium (LD) among all candidate loci was calculated using ldx, a package which uses an approximate maximum‐likelihood approach from pooled resequencing data (Feder et al., 2012). LD was calculated as *r* ^2^, the square of the correlation between alleles of SNP pairs within the paired sequence reads of each population. We subsequently calculated the average LD for each pairwise SNP comparison across sample sites. Next, we assessed the distribution of candidate loci and signatures of selection across the reference *H*. *rubra* genome consisting of 2854 annotated scaffolds varying between 1830 and 1.1 × 10^7^ bp in length (Gan et al., 2019). This was achieved by regressing the total number of candidate loci against scaffold length using the ggpubr package for R (Kassambara & Kassambara, 2020). Scaffolds with exceptionally large numbers of candidate loci relative to scaffold length (deviating from a linear distribution) were interrogated further using the package ldblockshow (Dong et al., 2021) to measure pairwise linkage disequilibrium and haplotype blocks using the default ‐SeleVar option to calculate *D*′ (the ratio of the difference between the observed and expected frequency of a haplotype, and its maximum value when considering total allele frequencies).

snpeff version 2.0.3 (Cingolani et al., 2012) was used to map candidate SNP loci to the *H*. *rubra* genome and predict variant impacts: high (highly disruptive impact on protein function), moderate (nonsynonymous mutations, possible change in protein effectiveness), low (unlikely to change protein behaviour) or a modifier (synonymous mutations, noncoding or intergenic variant). Functional classification of candidate genes was achieved by aligning the peptide sequences for mapped candidate *H*. *rubra* genes with the annotated genomes for human (NCBI RefSeq IDs NC_000001–NC_000024), pacific oyster (RefSeq IDs NC_047559–NC_047568), scallop (RefSeq ID NC_007234.1) and the blue mussel (RefSeq ID NC_006161.1) using diamond (Buchfink et al., 2015). A maximum e‐value of 1e^−40^ was set to conservatively estimate the likelihood of similar gene functions between taxonomies. Protein GI accessions from the top hit of diamond alignments were imported into the web‐based version of the david bioinformatics tool (Huang et al., 2009a, 2009b), where corresponding annotations were generated. Given the challenges of functional annotations when dealing with large numbers of loci, we took the conservative approach of annotating only gene homologues known to be associated with virus–host interactions, in particular herpesvirus response pathways, including those in response to HaHV‐1 in the AVG‐resistant *H*. *iris*.

## 3 RESULTS

### 3.1 Genotyping and overall population structure

**FIGURE 2:**
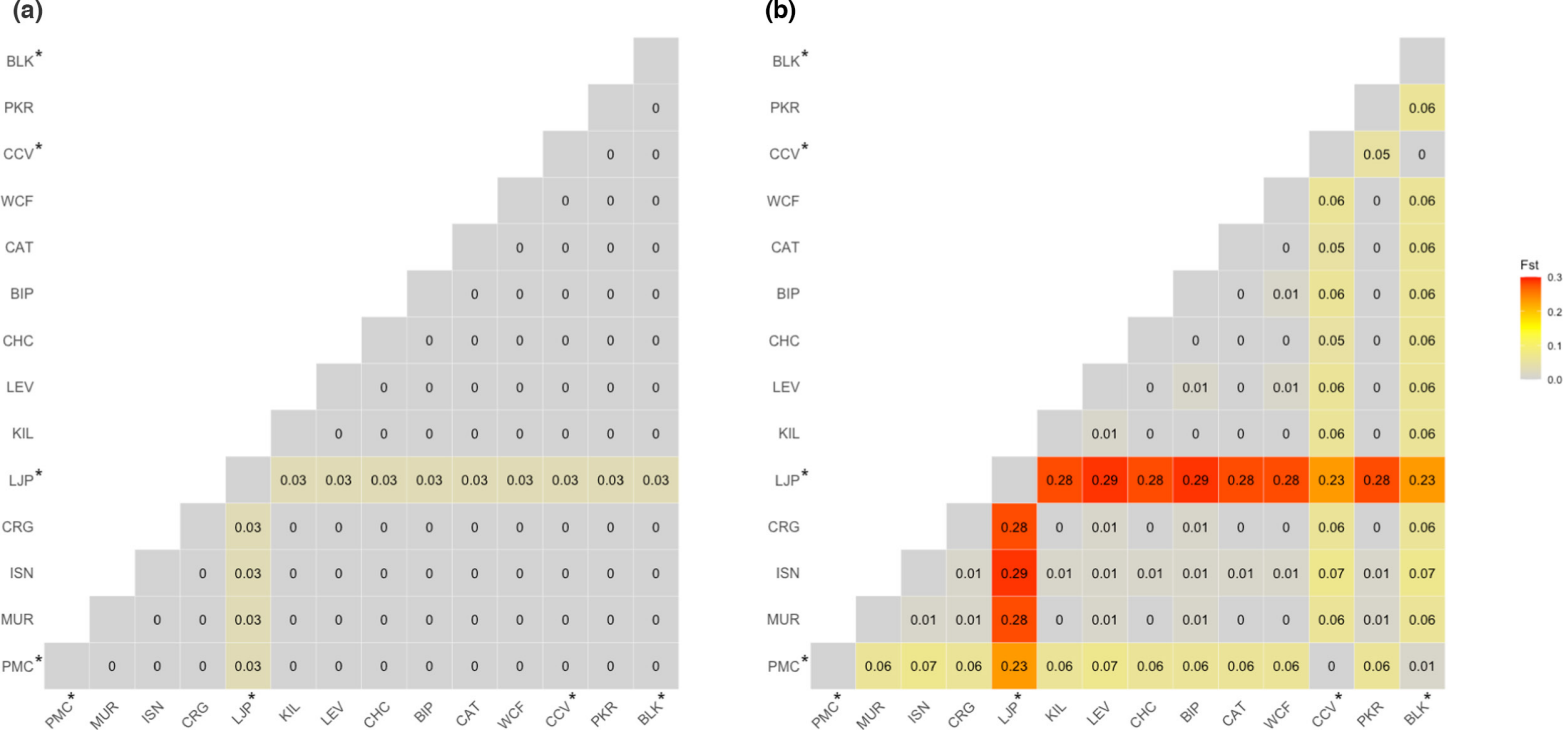
Heatmap of pairwise estimates of genetic differentiation (*F*_ST_) among sample locations based on (a) all 7,745,655 SNPs and (b) the 25,854 SNP loci associated with AVG exposure. *Virus‐unaffected sample locations

Pooled whole genome resequencing of 384 *Haliotis rubra* specimens from 14 locations yielded a total of 7,745,655 SNPs that were used for population genomic analyses. Estimates of overall genetic structure indicated a lack of structure and panmixia across the sampling distribution. Specifically, global *F*_ST_ did not differ significantly from zero (*F*_ST_ = 0.00, *p* > .05), consistent with reports of panmixia from previous population genomic studies on *H*. *rubra* (Miller et al., 2016, 2019). Additionally, no pairwise estimate of *F*_ST_ between sampling locations was found to differ significantly from zero (Figure 2a). While some sample locations included in this study were chosen based on modelled dependencies on self‐recruitment, our results and those of Miller et al. (2016, 2019) indicate that there is sufficient gene flow across all locations to supress signatures of genetic structure.

### 3.2 Genotype × environment association analysis

**FIGURE 3:**
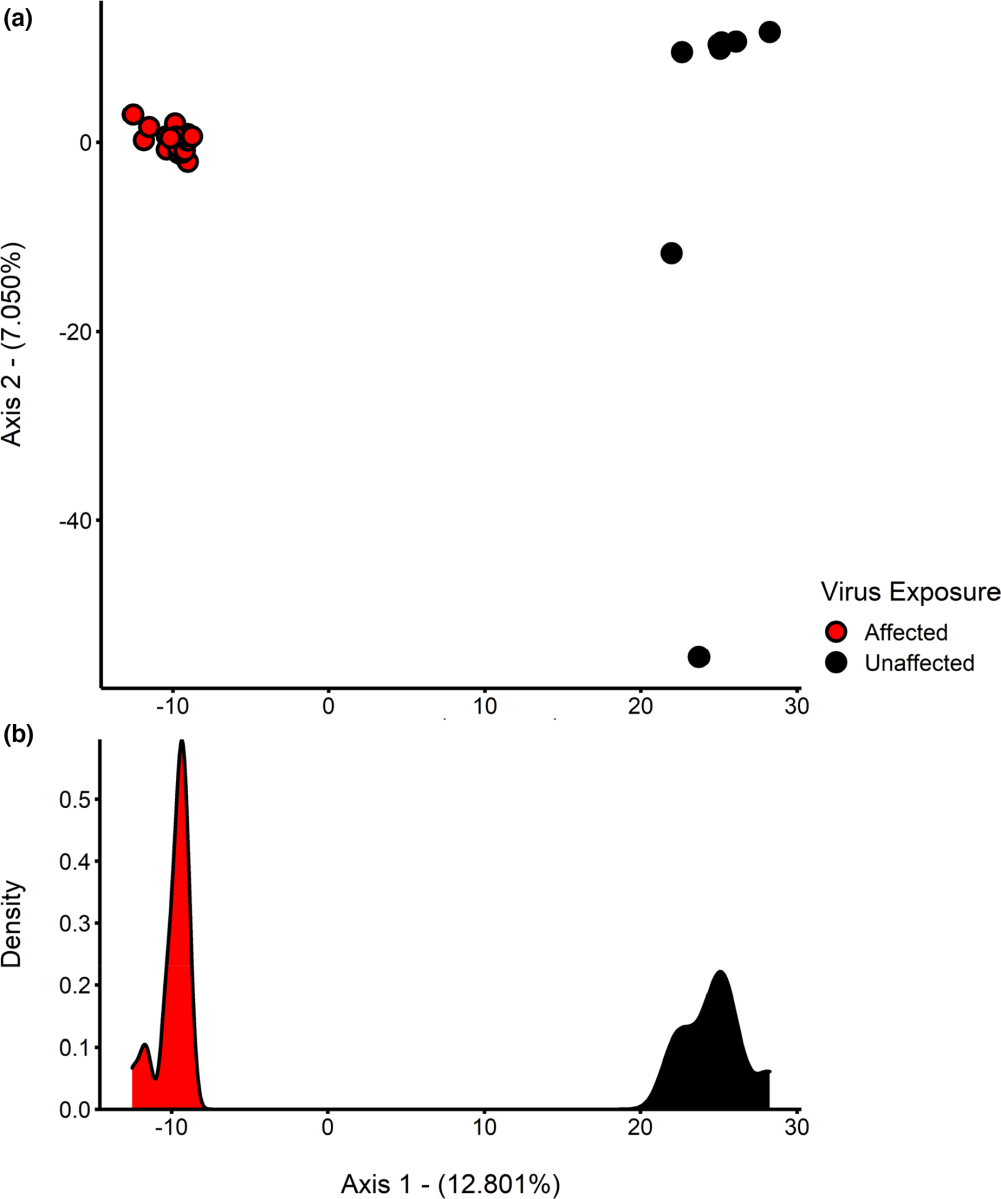
Plots of eigenvalues from the principal components analysis: (a) plot of axis 1 and 2 eigenvalues, and (b) density plot of axis 1 eigenvalues. Plots are based on candidate SNP genotypes from each of the 28 pooled whole genome resequencing libraries representing virus‐affected (red) and unaffected (black) fishing stocks

Our genome scans found 25,854 candidate SNPs with strong associations with virus exposure (BF > 50), with the PCA based on candidate loci revealing clear patterns of genetic structuring between locations varying in historical AVG exposure (Figure 3). Estimates of *F*_ST_ based on candidate loci also indicated significant genetic structure among sample locations (*F*_ST_ = 0.06, *p* < .05), with pairwise estimates suggesting significant genetic structure between virus‐affected and ‐unaffected sites (Figure 2b). All pairwise estimates of *F*_ST_ including a single virus‐unaffected location (LJP) were significantly different from zero, but estimates involving this location and other unaffected locations were notably weaker. To account for potential distorting effects associated with LJP, association analyses were repeated including all sites except LJP. Repeat analyses still recovered 21,039 candidate SNPs, with a PCA based on these candidate loci revealing consistent patterns of genetic structuring between locations varying in historical AVG exposure (Figure S1).

**FIGURE 4:**
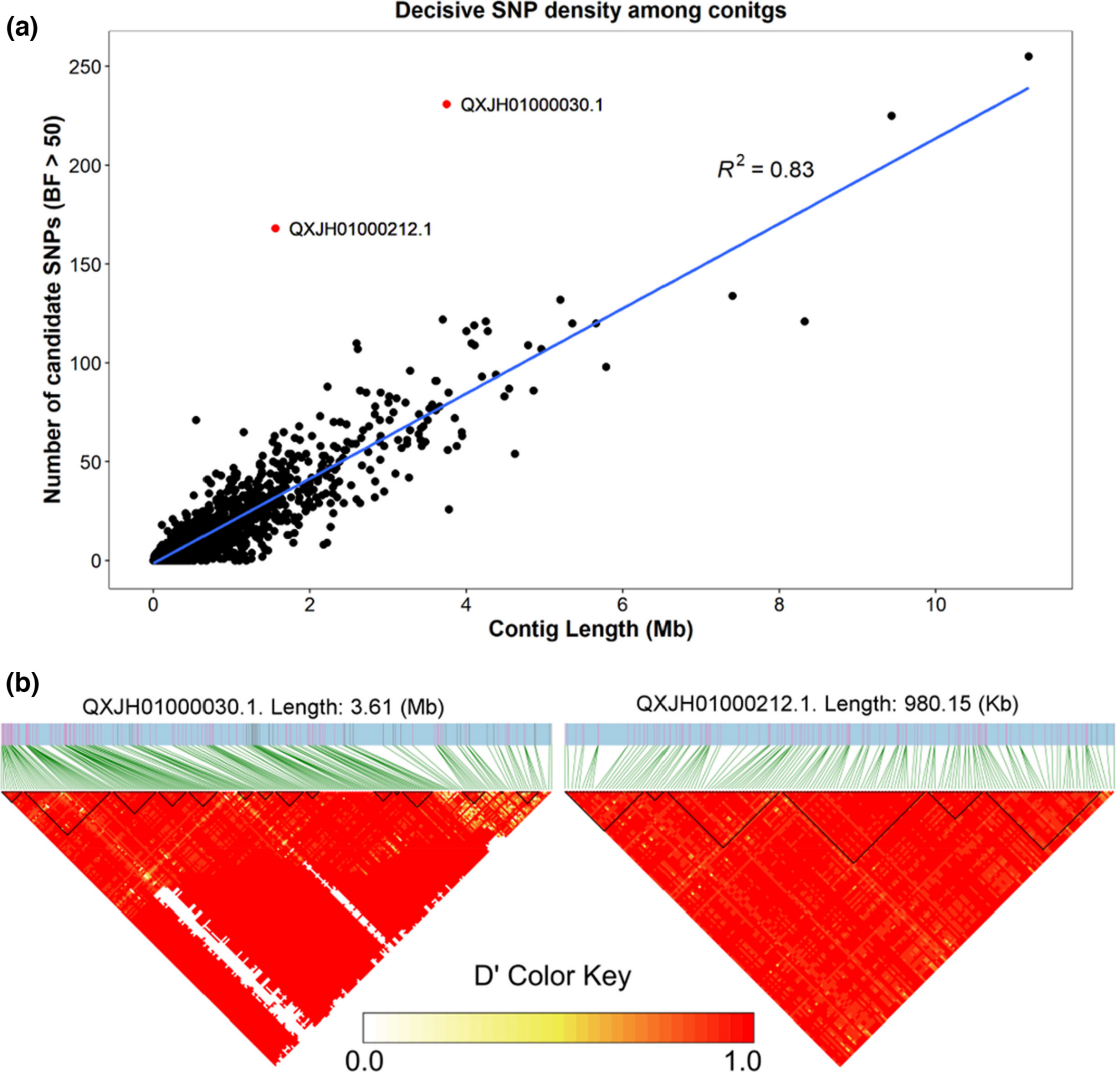
(a) Regression analysis indicating a positive linear relationship between number of candidate SNPs (BF > 50) and scaffold length. Outlier scaffolds with a greater frequency of candidate SNPs relative to scaffold length are plotted in red. (b) Linkage disequilibrium heatmaps of scaffolds with the greatest number of candidate SNPs (QXH01000030.1 and QXJH01000212.1) generated with the package ldblockshow (Dong et al., 2021). Heatmaps depict the pairwise linkage disequilibrium measure of *D*′ (refer to colour key) between each SNP with a BF ≥ 50, while green lines link the relative position of the candidate SNPs to the heatmap. In addition, black triangle sections represent detected haplotype blocks; these are genomic regions of low recombination

Estimates of LD were high at all locations (mean *r* ^2^ = .61 ± 0.01 *SD*) indicating nonrandom association of alleles, while comparisons of *r* ^2^ between affected and unaffected stocks did not differ significantly (*p* > .05). Analyses suggest a genome‐wide pattern, with regression analyses indicating a strong linear relationship between number of candidate loci and scaffold length (*R* ^2^ = .83; Figure 4a). However, scaffolds QXJH01000030.1 and QXJH01000212.1 exhibited a higher number of candidate loci relative to scaffold length (deviating from the linear distribution), with candidate SNPs comprising 0.0062% and 0.0108% of the total scaffold nucleotides, respectively. Pairwise linkage among SNPs across the entirety of these scaffolds was high (*D*′ ≈ 1), with the detection of large haplotype blocks indicating large sections of the genome linked to virus exposure (Figure 4b).

### 3.3 Functional annotations

SNP loci showing significant associations with virus exposure were successfully mapped to the annotated *H*. *rubra* genome. snpeff analyses predicted 333 candidate loci to have moderate effect on protein function (involving nonsynonomous mutations), while 489 candidates were predicted to have low effect, and 24,722 candidates were recognized as noncoding or intergenic variants. Candidate loci that successfully mapped to *H*. *rubra* genome peptide sequences were found to correspond with gene homologues in other animal systems including haliotids, nonhaliotid marine molluscs, crustaceans and humans. These include 13 gene homologues linked to HaHV‐1 immunity in New Zealand pāua (*Haliotis iris*) and 13 genes associated with herpes virus response pathways in Japanese disk abalone (*Haliotis discus hannai*), decapod crustaceans (*Penaues monodon* and *Procambarus clarkia*) and humans (Table 2). An additional 10 peptides mapped to gene homologues associated with host–virus interactions in various haliotids (*H*. *discus hannai*,*H*. *laevigata* and *H*. *ruscefens*), decapod crustaceans (*Pe*. *monodon* and *Pr*. *clarkia*) and humans (Table 2). All gene homologues and known functions are provided in Table 2. Notable findings include several genes linked to chitin‐binding peritrophin‐A domain, and the cytochrome P450 (CYP) 3A family, which have recognized associations with immune responses in aquatic molluscs (Badariotti et al., 2007; Zhao et al., 2017) and humans (Fattahi et al., 2018), respectively. Also, the CREB‐binding protein (CBP) was identified, which is associated with herpesvirus responses in humans (Chen et al., 2020; Gwack et al., 2001), as well as immune pathways for C‐type lectins that are important contributors to innate immune responses in invertebrates (Nam et al., 2016; Qin et al., 2019; Zhang et al., 2018).

**TABLE 2:** List of predicted genetic variant impacts, and genes that candidate loci mapped to. The table also includes gene functions, as well as the species from which these functions have been reported and their respective references

Number of candidate SNPs	Predicted variant impact	Associated gene	Associated gene function	Species	Reference(s)
Genes involved *H*. *iris* HaHV−1 immune response					
17	Moderate, low, modifier	SLC1A2	Excitatory amino acid transporter 2, response to HaHV−1 exposure	*Haliotis iris*	Neave et al., 2019
35	Moderate, low, modifier	CYP3A4	Response to HaHV−1 exposure	*Haliotis iris*	Neave et al., 2019
6	Moderate, modifier	ACE	Response to HaHV−1 exposure	*Haliotis iris*	Neave et al., 2019
2	Modifier	POU6F2	Response to HaHV−1 exposure	*Haliotis iris*	Neave et al., 2019
3	Modifier	NLGN4X	Response to HaHV−1 exposure	*Haliotis iris*	Neave et al., 2019
2	Modifier	CYP3A7	Response to HaHV−1 exposure	*Haliotis iris*	Neave et al., 2019
1	Modifier	Peritrophin 44 like (LOC105317660)	Chitin‐binding peritrophin‐A domain, response to HaHV−1 exposure	*Haliotis iris*	Neave et al., 2019
1	Modifier	Uncharacterized LOC105326593 (LOC105326593)	Chitin‐binding peritrophin‐A domain, response to HaHV−1 exposure	*Haliotis iris*	Neave et al., 2019
17	Modifier	CHIT1	Chitin‐binding peritrophin‐A domain, response to HaHV−1 exposure	*Haliotis iris*	Neave et al., 2019
15	Modifier	CYP3A5	Response to HaHV−1 exposure	*Haliotis iris*	Neave et al., 2019
1	Modifier	CYP3A43	Response to HaHV−1 exposure	*Haliotis iris*	Neave et al., 2019
14	Modifier	Ganglioside GM2 activator like (LOC105346019)	Chitin‐binding peritrophin‐A domain, response to HaHV−1 exposure	*Haliotis iris*	Neave et al., 2019
1	Modifier	Ganglioside GM2 activator like (LOC105348613)	Chitin‐binding peritrophin‐A domain, response to HaHV−1 exposure	*Haliotis iris*	Neave et al., 2019
Gene homologues associated with herpesvirus response pathways					
2	Modifier	CASP8	Herpesvirus response pathway	*Haliotis discus hannai*	Nam et al., 2016
1	Modifier	TAF10	Herpesvirus response pathway	*Homo sapiens*	Wagner & DeLuca, 2013
1	Modifier	ARNTL	Herpesvirus response pathway	*Homo sapiens*	Edgar et al., 2016
7	Modifier	EEF1D	Herpesvirus response pathway	*Homo sapiens*	Boulben et al., 2003
2	Modifier	SRSF7	Herpesvirus response pathway	*Homo sapiens*	Tang et al., 2019
6	Modifier	Ube2r2	Herpesvirus response pathway	*Homo sapiens*	Beard et al., 2015
2	Modifier	CREBBP	Herpesvirus response pathway, C‐type lectin containing or involved in c‐type lectin pathway (invertebrate immune response)	*Haliotis discus hannai*,*Procambarus clarkii*,*Penaeus monodon*	Nam et al., 2016, Zhang et al., 2018, Qin et al., 2019, Wang & Wang, 2013
2	Modifier	SRPK1	Herpesvirus response pathway	*Homo sapiens*	Souki & Sandri‐Golden, 2009
2	Modifier	TAF6	Herpesvirus response pathway	*Homo sapiens*	Wagner & DeLuca, 2013
1	Modifier	TAB1	Herpesvirus response pathway	*Homo sapiens*	Jahanban‐Esfahlan et al., 2019
1	Modifier	Csnk2b	Herpesvirus response pathway	*Homo sapiens*	Carter, 2011
4	Modifier	Hnrnpk	Herpesvirus response pathway	*Homo sapiens*	Schmidt et al., 2010
1	Modifier	PPP1CA	Herpesvirus response pathway	*Homo sapiens*	Silva et al., 2015
Gene homologues associated with host–virus interactions					
4	Modifier	HIST1H2AA	Abalone immune response	*Haliotis discus hannai*	Nam et al., 2016
1	Low	COTL1	Tropomyosin, abalone immune response	*Haliotis discus hannai*	Nam et al., 2016
1	Low, modifier	PDIA3	Protein disulphide isomerase activity, abalone immune response	*Haliotis discus hannai*	Nam et al., 2016
1	Modifier	HSP90AB1	Abalone immune response	*Haliotis discus hannai*	Nam et al., 2016
4	Modifier	Histone H2A (LOC105320412)	Abalone immune response	*Haliotis discus hannai*	Nam et al., 2016
8	Low, modifier	AVIL	Gelsolin domain, abalone immune response	*Haliotis discus hannai*	Nam et al., 2016
1	Modifier	Hsp70 member 12A (HSPA12A)	Abalone immune response	*Haliotis laevigata*,*Haliotis ruscefens*,*Haliotis discus hannai*	Shiel et al., 2015, Brokordt et al. 2015, Nam et al., 2016
3	Modifier	PSMA8	C‐type lectin containing or involved in c‐type lectin pathway (invertebrate immune response)	*Haliotis discus hannai*,*Procambarus clarkii*,*Penaeus monodon*	Nam et al., 2016, Zhang et al., 2018, Qin et al., 2019, Wang & Wang, 2013
1	Modifier	IRAK4	Toll‐like receptor activity, innate immunity	*Crassostrea gigas*,*Haliotis diversicolor*	Tang et al., 2017, Ge et al., 2011
2	Modifier	TIA1	Viral translation inhibition	*Homo sapiens*	McCormick & Khaperskyy, 2017

## 4 DISCUSSION

We provide evidence of rapid genetic changes in as little as a single generation in wild *Haliotis rubra* populations decimated by HaHV‐1. Specifically, our genome scans identified SNP loci associated with virus exposure, many of which mapped to genes known to contribute to HaHV‐1 immunity, herpes virus response pathways, and host–virus interactions in haliotids and other animal systems. These findings require experimental validation but are consistent with rapid evolutionary changes in *H*. *rubra* fishing stocks impacted by disease, with stock recovery potentially influenced by evolved resistance. This study highlights the pace at which selection can act to counter disease in wildlife communities by leading to an increased frequency of potentially adaptive genotypes. The implications of these findings are discussed in the context of future infectious diseases management in abalone fisheries and wildlife conservation more generally.

### 4.1 Evidence of rapid evolution in *H. rubra*

Our analyses identified 25,854 SNP loci associated with AVG exposure in *H*. *rubra*. Multiple lines of evidence point to selection being responsible for driving specific genetic variants to higher frequencies in virus‐affected populations, rather than random demographic factors such as bottleneck effects. Random demographic factors are capable of causing shifts in allele frequencies (particularly rare alleles) in populations that have suffered major declines, depending on the number of surviving individuals and the influence of random genetic drift in subsequent generations (Willi et al., 2022). However, the number of surviving abalone at virus‐impacted locations still consisted of many 100s to 1000s of individuals (Western Abalone Divers Association, 2020) expected to maintain genetic diversity. Also, we have shown that the observed patterns of differentiation between virus‐affected and unaffected fishing stocks are not being driven by rare alleles (Figure S2). Additionally, opportunities for allele frequencies to be influenced by random genetic drift are limited given the animals included in this study are either survivors or F_1_/F_2_ progeny of those survivors. Furthermore, we observed consistent genetic changes across a spatially replicated and stratified sampling distribution, which are unlikely to arise randomly in the absence of selection. Finally, we provide evidence of functional associations of candidate SNPs to genes known to contribute to disease adaptation in sister haliotid taxa, including a number of genes that contribute to HaHV‐1 immunity.

Despite the large number of candidate loci found to be associated with AVG exposure, it is likely that only some are directly influenced by selection and potentially contributing to AVG immunity. Evidence of LD between loci, and the noncoding and intergenic nature of most SNPs indicates many indirect associations due to physical linkage with adaptive loci that are embedded in large haploblocks and responding to selection (Uffelmann et al., 2021). Further, it is possible that some candidate loci are in statistical linkage, sharing similar allele frequency distributions across haploblocks and chromosomes (i.e. genetic indistinguishability; Skelly et al., 2016), and inflating the overall number of candidate loci. Despite the potential for linkage, a strong linear relationship between genome scaffold length and density of candidate SNPs was observed, suggesting a genome‐wide response to virus exposure. Analyses indicate a higher incidence of candidate SNPs on two scaffolds, which could represent important gene‐rich adaptive regions (Hohenlohe et al., 2012). However, further work is needed to test their functional significance as the genes annotated within these regions are not known to contribute to virus resistance. Nevertheless, our analyses indicate that some candidate loci may be directly involved in disease adaptation in *H*. *rubra*.

In particular, functional annotations of candidate loci point to associations with genes and protein domains that contribute to HaHV‐1 immunity in the New Zealand pāua (*Haliotis iris*). Neave et al. (2019) first characterized genes associated with HaHV‐1 immunity in *H*. *iris* through transcriptome analyses of animals subject to HaHV‐1 immersion challenge tests. Their study was the first to characterize the molecular basis of HaHV‐1 immunity in a haliotid species, and our study complements these findings by identifying a common set of genes involved in haliotid host response to HaHV‐1 exposure. Functional annotations of candidate loci also point to associations with genes and protein domains contributing to herpes simplex virus responses and immune responses in various haliotids and other animal systems. Importantly, our analyses indicate that a large number of candidate loci involve nonsynonymous mutations that are expected to have an effect on protein structure and function. It is also possible that some candidate SNPs recognized as being noncoding and intergenic variants might provide functionally important regulatory roles in genomic processes (Gusev et al., 2014; Li et al., 2012; Wei et al., 2020). Overall, these findings strongly support the notion that divergent adaptation, involving a polygenic response and possible selection for disease resistance phenotypes, has occurred in *H*. *rubra* fishing stocks impacted by AVG.

The results of this study point to rapid genetic changes in *H*. *rubra* fishing stocks impacted by disease. However, experimental validation will be needed to link any genetic changes to disease resistance. Challenge tests involving the exposure of animals with putatively resistant genotypes to HaHV‐1 will help to determine if, and how much, HaHV‐1 resistance is determined by the candidate genotypes (Corbeil et al., 2016; Crane et al., 2013). Although previous challenge tests performed by Crane et al. (2013) showed no, or very low, resistance to HaHV‐1 in *H*. *rubra*, the animal source locations differ from those included in our study. This points to potential spatial variation in adaptive responses across the fishery, and possible genotype–environment interactions affecting the expression of resistant phenotypes in different environmental settings (Hoffmann et al., 2020). Nevertheless, a large number of loci appear to be responding to the virus in our study, suggesting that changes in resistance will represent a polygenic response that can be followed by controlled breeding studies (Guarna et al., 2017; Gutierrez et al., 2018). The response to selection in these studies will depend on levels of heritable variation as well as the intensity of selection, which will determine the impact of the phenotypic change. Breeding studies and experimental trials will also be essential to assess trait heritability and genotype–environment interactions, particularly if industry intends to control for resistance traits in a culture environment for breeding purposes (discussed below).

### 4.2 Implications for fisheries management

Re‐emergence of AVG remains a significant threat to the economic viability of *H*. *rubra* fisheries in southeastern Australia (Corbeil, 2020; Lafferty et al., 2015). Therefore, characterizing the spatial distribution and prevalence of disease‐resistant genotypes will help managers identify stocks expected to be either resilient or vulnerable to AVG re‐emergence. Previous population genomic research has indicated a lack of biological stock structure in these fisheries (Miller et al., 2016), suggesting that gene flow could contribute to the spread of adaptive genotypes and resilience of naïve fishing stocks. Gene flow from unaffected parts of the fishery could also eventually reduce the frequency of adaptive genotypes over time in the absence of ongoing selection, but such effects are yet to become apparent. Whether selection for resistance will be a recurring process is still unclear, although the recent outbreak recorded in 2021 at several fishing locations previously impacted by AVG in the early 2000s indicates that this is a possibility. Notably, the recent outbreak has resulted in minimal animal mortality and disease spread, supporting the notion of evolved resistance following previous exposure to disease. Contrasting the genetic profiles of survivors and those that have succumbed to the virus at these locations as a result of the most recent outbreak will help to reinforce the findings of the current study. Similarly, the recent outbreak provides a unique opportunity to genomically select putatively resistant and vulnerable animals for challenge tests aimed at providing functional validation of AVG resistance.

Evidence of panmixia in *H*. *rubra* (Miller et al., 2016) suggests that standing genetic variation is likely to persist within disease‐naïve populations allowing for *in situ* adaptation to HaHV‐1. However, strategic stock augmentation activities, involving the translocations of animals with AVG‐resistant genotypes, could potentially assist the spread of genotypes to reduce risks of vulnerability across wild fisheries. Also, there may be future opportunities to biosecure farm fisheries through the establishment of AVG‐resistant breeding programmes, similar to disease‐related breeding programmes in other farmed mollusc, crustacean and finfish fisheries around the world (Kjøglum et al., 2008; Moss et al., 2012; Potts et al., 2021; Ragone Calvo et al., 2003). Overall, these results add to those of Miller et al. (2019) demonstrating patterns of genetic adaptation across environmental gradients and the adaptability of *H*. *rubra* populations to new environmental conditions. This is pertinent in southeastern Australia where rapid changes in the physical marine climate are threatening commercial fisheries through shifts in species distributions (Johnson et al., 2011; Ling, 2008), changes in habitat and trophic interactions (Holland et al., 2021), and risks of infectious diseases (Oliver et al., 2017).

## 5 CONCLUSIONS

While it has been proposed that selection in some species may fail to keep pace with rates of pathogen evolution and the emergence and turnover of novel diseases (Hawley et al., 2013; Ujvari et al., 2014), our study demonstrates that rapid evolutionary responses within a single generation may be possible in large populations under extreme selective pressure. This study highlights the value of genome scans for identifying signatures of potential adaptation among natural populations differing in virus exposure and characterizing putative genes that contribute to disease‐resistant phenotypes. Future studies of this nature will be critical for understanding the potential for rapid evolution in other species threatened by infectious diseases, informing the management of new outbreaks, and securing populations with little or no resistance.

## AUTHOR CONTRIBUTIONS

This project was conceived by A.D.M., while O.J.H., M.T., A.D.M. and L.C. were responsible for generating and analysing the data, with assistance from C.A. and A.A.H. Writing of the manuscript was led by O.J.H. and A.D.M. with assistance from all authors.

## CONFLICT OF INTEREST

The authors declare no conflict of interest.

### OPEN RESEARCH BADGES

This article has earned an Open Data Badge for making publicly available the digitally‐shareable data necessary to reproduce the reported results. The data is available at https://doi.org/10.5061/dryad.b5mkkwhfb.

## Supporting information

Fig S1‐2

## Data Availability

VCF files containing all filtered SNPs and candidate loci, data sets used for baypass and snpeff analyses, and details of all annotated SNP loci are deposited on DataDryad (https://doi.org/10.5061/dryad.b5mkkwhfb). Raw data (BAM format) are available upon request.

## References

[mec16499-bib-0001] Agriculture Victoria . (2021) Abalone Disease. Accessed July 2021. https://agriculture.vic.gov.au/biosecurity/animal‐diseases/abalone‐disease

[mec16499-bib-0002] Andrew, N. (1999). Under southern seas: The ecology of Australia's rocky reefs. UNSW Press.

[mec16499-bib-0003] Auteri, G. G. , & Knowles, L. L. (2020). Decimated little brown bats show potential for adaptive change. Scientific Reports, 10, 3023. 10.1038/s41598-020-59797-4 32080246 PMC7033193

[mec16499-bib-0004] Badariotti, F. , Lelong, C. , Dubos, M. P. , & Favrel, P. (2007). Characterization of chitinase‐like proteins (Cg‐Clp1 and Cg‐Clp2) involved in immune defence of the mollusc Crassostrea gigas. FEBS Journal, 274, 3646–3654. 17608806 10.1111/j.1742-4658.2007.05898.x

[mec16499-bib-0005] Bai, C.‐M. , Li, Y.‐N. , Chang, P.‐H. , Jiang, J.‐Z. , Xin, L.‐S. , Li, C. , Wang, J.‐Y. , & Wang, C.‐M. (2019). Susceptibility of two abalone species, Haliotis diversicolor supertexta and Haliotis discus hannai, to Haliotid herpesvirus 1 infection. Journal of Invertebrate Patholology, 160, 26–32. 10.1016/j.jip.2018.11.008 30513284

[mec16499-bib-0006] Bai, C. M. , Zhang, S. M. , Li, Y. N. , Xin, L. S. , Rosani, U. , & Wang, C. M. (2019). Dual transcriptomic analysis reveals a delayed antiviral response of *Haliotis diversicolor supertexta* against Haliotid Herpesvirus‐1. Viruses, 11, 383. 10.3390/v11040383 PMC6520846 31022987

[mec16499-bib-0100] Beard, J. A. , Tenga, A. , & Chen, T. (2015). The interplay of NR4A receptors and the oncogene–tumor suppressor networks in cancer. Cellular Signalling, 27(2), 257–266. 10.1016/j.cellsig.2014.11.009 25446259 PMC4276441

[mec16499-bib-0007] Berger, L. , Speare, R. , Daszak, P. , Green, D. E. , Cunningham, A. A. , Goggin, C. L. , Slocombe, R. , Ragan, M. A. , Hyatt, A. D. , McDonald, K. R. , Hines, H. B. , Lips, K. R. , Marantelli, G. , & Parkes, H. (1998). Chytridiomycosis causes amphibian mortality associated with population declines in the rain forests of Australia and Central America. Proceedings of the National Academy of Sciences of the United States of America, 95, 9031–9036. 10.1073/pnas.95.15.9031 9671799 PMC21197

[mec16499-bib-0008] Blanchong, J. A. , Robinson, S. J. , Samuel, M. D. , & Foster, J. T. (2016). Application of genetics and genomics to wildlife epidemiology. Journal of Wildlife Management, 80, 593–608. 10.1002/jwmg.1064

[mec16499-bib-0009] Bolger, A. M. , Lohse, M. , & Usadel, B. (2014). Trimmomatic: A flexible trimmer for Illumina sequence data. Bioinformatics, 30, 2114–2120. 10.1093/bioinformatics/btu170 24695404 PMC4103590

[mec16499-bib-0010] Bonhomme, M. , Chevalet, C. , Servin, B. , Boitard, S. , Abdallah, J. , Blott, S. , & SanCristobal, M. (2010). Detecting selection in population trees: the Lewontin and Krakauer test extended. Genetics, 186(1), 241–262. 10.1534/genetics.110.117275 20855576 PMC2940290

[mec16499-bib-0011] Bonneaud, C. , Balenger, S. L. , Russell, A. F. , Zhang, J. , Hill, G. E. , & Edwards, S. V. (2011). Rapid evolution of disease resistance is accompanied by functional changes in gene expression in a wild bird. Proceedings of the National Academy of Sciences of the United States of America, 108, 7866–7871. 10.1073/pnas.1018580108 21525409 PMC3093480

[mec16499-bib-0012] Bosch, J. , Fernandez‐Beaskoetxea, S. , Garner, T. W. J. , & Carrascal, L. M. (2018). Long‐term monitoring of an amphibian community after a climate change‐ and infectious disease‐driven species extirpation. Global Change Biology, 24, 2622–2632. 10.1111/gcb.14092 29446515

[mec16499-bib-0097] Boulben, S. , Monnier, A. , Le Breton, M. , Morales, J. , Cormier, P. , Belle, R. , & Mulner‐Lorillon, O. (2003). Sea urchin elongation factor 1δ (EF1δ) and evidence for cell cycle‐directed localization changes of a sub‐fraction of the protein at M phase. Cellular and Molecular Life Sciences CMLS, 60(10), 2178–2188. 14618264 10.1007/s00018-003-3201-x PMC11138503

[mec16499-bib-0013] Brearley, G. , Rhodes, J. , Bradley, A. , Baxter, G. , Seabrook, L. , Lunney, D. , Liu, Y. , & McAlpine, C. (2013). Wildlife disease prevalence in human‐modified landscapes. Biological Reviews, 88, 427–442. 10.1111/brv.12009 23279314

[mec16499-bib-0109] Brokordt, K. B. , González, R. C. , Farías, W. J. , & Winkler, F. M. (2015). Potential response to selection of HSP70 as a component of innate immunity in the abalone Haliotis rufescens. PLoS One, 10(11), e0141959. 10.1371/journal.pone.0141959 26529324 PMC4631488

[mec16499-bib-0014] Buchfink, B. , Xie, C. , & Huson, D. H. (2015). Fast and sensitive protein alignment using DIAMOND. Nature Methods, 12, 59–60. 10.1038/nmeth.3176 25402007

[mec16499-bib-0105] Carter, C. J. (2011). Alzheimer's disease plaques and tangles: Cemeteries of a pyrrhic victory of the immune defence network against herpes simplex infection at the expense of complement and inflammation‐mediated neuronal destruction. Neurochemistry International, 58(3), 301–320. 10.1016/j.neuint.2010.12.003 21167244

[mec16499-bib-0015] Cawthorn, R. J. (2011). Diseases of American lobsters (*Homarus americanus*): A review. Journal of Invertebrate Pathology, 106, 71–78. 10.1016/j.jip.2010.09.010 21215356

[mec16499-bib-0016] Chang, P. H. , Kuo, S. H. , Lai, S. H. , Yang, H. S. , Ting, Y. Y. , Hsu, C. L. , & Chen, H. C. (2005). Herpes‐like virus infection causing mortality of cultured abalone *Haliotis diversicolor supertexta* in Taiwan. Diseases of Aquatic Organisms, 65, 23–27. 10.3354/dao065023 16042040

[mec16499-bib-0017] Chen, C. , Feng, P. , & Slots, J. (2020). Herpesvirus‐bacteria synergistic interaction in periodontitis. Periodontology, 2000(82), 42–64. 10.1111/prd.12311 PMC7382446 31850623

[mec16499-bib-0018] Cingolani, P. , Platts, A. , Wang, L. L. , Coon, M. , Nguyen, T. , Wang, L. , Land, S. J. , Lu, X. , & Ruden, D. M. (2012). A program for annotating and predicting the effects of single nucleotide polymorphisms, SnpEff: SNPs in the genome of *Drosophila melanogaster* strain w1118; iso‐2; iso‐3. Fly (Austin), 6, 80–92. 10.4161/fly.19695 22728672 PMC3679285

[mec16499-bib-0019] Clemente, S. , Lorenzo‐Morales, J. , Mendoza, J. C. , López, C. , Sangil, C. , Alves, F. , Kaufmann, M. , & Hernández, J. C. (2014). Sea urchin Diadema africanum mass mortality in the subtropical eastern Atlantic: Role of waterborne bacteria in a warming ocean. Marine Ecology Progress Series, 506, 1–14. 10.3354/meps10829

[mec16499-bib-0020] Corbeil, S. (2020). Abalone Viral Ganglioneuritis. Pathogens, 9, 720. 10.3390/pathogens9090720 PMC7558354 32882932

[mec16499-bib-0021] Corbeil, S. , McColl, K. A. , Williams, L. M. , Slater, J. , & Crane, M. S. (2017). Innate resistance of New Zealand paua to abalone viral ganglioneuritis. Journal of Invertebrate Pathology, 146, 31–35. 10.1016/j.jip.2017.04.005 28431886

[mec16499-bib-0022] Corbeil, S. , Williams, L. , McColl, K. , & Crane, M. (2016). Australian abalone (Haliotis laevigata, H. rubra and H. conicopora) are susceptible to infection by multiple abalone herpesvirus genotypes. Diseases of Aquatic Organisms, 119, 101–106. 10.3354/dao02989 27137068

[mec16499-bib-0023] Crane, M. S. J. , Corbeil, S. , Williams, L. M. , McColl, K. A. , & Gannon, V. (2013). Evaluation of abalone viral ganglioneuritis resistance among wild abalone populations along the Victorian coast of Australia. Journal of Shellfish Research, 32, 67–72. 10.2983/035.032.0112

[mec16499-bib-0024] Crosson, L. M. , Lottsfeldt, N. S. , Weavil‐Abueg, M. E. , & Friedman, C. S. (2020). Abalone withering syndrome disease dynamics: Infectious dose and temporal stability in seawater. Journal of Aquatic Animal Health, 32, 83–92. 10.1002/aah.10102 32339356

[mec16499-bib-0025] Cunningham, A. A. , Daszak, P. , & Wood, J. L. N. (2017). One Health, emerging infectious diseases and wildlife: Two decades of progress? Philosophical Transactions of the Royal Society B‐Biological Sciences, 372, 20160167. 10.1098/rstb.2016.0167 PMC5468692 28584175

[mec16499-bib-0026] Daszak, P. , Cunningham, A. A. , & Hyatt, A. D. (2000). Wildlife ecology ‐ Emerging infectious diseases of wildlife ‐ Threats to biodiversity and human health. Science, 287, 443–449. 10.1126/science.287.5452.443 10642539

[mec16499-bib-0027] Dong, S. S. , He, W. M. , Ji, J. J. , Zhang, C. , Guo, Y. , & Yang, T.‐L. (2021). LDBlockShow: a fast and convenient tool for visualizing linkage disequilibrium and haplotype blocks based on variant call format files. Briefings in Bioinformatics, 22(4), 1–6. 33126247 10.1093/bib/bbaa227

[mec16499-bib-0197] Edgar, R. S. , Stangherlin, A. , Nagy, A. D. , Nicoll, M. P. , Efstathiou, S. , O'Neill, J. S. , & Reddy, A. B. (2016). Cell autonomous regulation of herpes and influenza virus infection by the circadian clock. Proceedings of the National Academy of Sciences, 113(36), 10085–10090. 10.1073/pnas.1601895113 PMC5018795 27528682

[mec16499-bib-0028] Elbers, J. P. , Brown, M. B. , & Taylor, S. S. (2018). Identifying genome‐wide immune gene variation underlying infectious disease in wildlife populations ‐ a next generation sequencing approach in the gopher tortoise. Bmc Genomics, 19, 1–10. 29351737 10.1186/s12864-018-4452-0 PMC5775545

[mec16499-bib-0029] Epstein, B. , Jones, M. , Hamede, R. , Hendricks, S. , McCallum, H. , Murchison, E. P. , Schönfeld, B. , Wiench, C. , Hohenlohe, P. , & Storfer, A. (2016). Rapid evolutionary response to a transmissible cancer in Tasmanian devils. Nature Communications, 7, 12684. 10.1038/ncomms12684 PMC5013612 27575253

[mec16499-bib-0030] FAO FishStat (2021) Fisheries and Aquaculture Software. FishStat Plus – Universal Software for Fishery Statistical Time Series. FAO Fisheries and Aquaculture Department, Rome. Accessed July 2021. http://www.fao.org/fishery

[mec16499-bib-0031] Fattahi, S. , Karimi Alivije, M. , Babamahmoodi, F. , Bayani, M. , Sadeghi Haddad Zavareh, M. , Asouri, M. , Lotfi, M. , Amirbozorgi, G. , & Akhavan‐Niaki, H. (2018). Cytochrome P450 genes (CYP2E1 and CYP1A1) variants and susceptibility to chronic hepatitis B virus infection. Indian Journal of Clinical Biochemistry, 33, 467–472. 10.1007/s12291-017-0698-6 30319195 PMC6170240

[mec16499-bib-0032] Feder, A. F. , Petrov, D. A. , & Bergland, A. O. (2012). LDx: Estimation of linkage disequilibrium from high‐throughput pooled resequencing data. PLoS One, 7, e48588. 23152785 10.1371/journal.pone.0048588 PMC3494690

[mec16499-bib-0033] Futschik, A. , & Schlotterer, C. (2010). The next generation of molecular markers from massively parallel sequencing of pooled DNA samples. Genetics, 186, 207–218. 10.1534/genetics.110.114397 20457880 PMC2940288

[mec16499-bib-0034] Gan, H. M. , Tan, M. H. , Austin, C. M. , Sherman, C. D. H. , Wong, Y. T. , Strugnell, J. , Gervis, M. , McPherson, L. , & Miller, A. D. (2019). Best foot forward: Nanopore long reads, hybrid meta‐assembly, and haplotig purging optimizes the first genome assembly for the Southern Hemisphere blacklip abalone (*Haliotis rubra*). Frontiers in Genetics, 10, 889. 10.3389/fgene.2019.00889 31608118 PMC6774278

[mec16499-bib-0035] Gautier, M. (2015). Genome‐wide scan for adaptive divergence and association with population‐specific covariates. Genetics, 201, 1555–1579. 10.1534/genetics.115.181453 26482796 PMC4676524

[mec16499-bib-0110] Ge, H. , Wang, G. , Zhang, L. , Zhang, Z. , Wang, S. , Zou, Z. , Yan, S. , & Wang, Y. (2011). Molecular cloning and expression of interleukin‐1 receptor‐associated kinase 4, an important mediator of Toll‐like receptor signal pathway, from small abalone Haliotis diversicolor. Fish & Shellfish Immunology, 30(4–5), 1138–1146. 10.1016/j.fsi.2011.02.018 21362486

[mec16499-bib-0036] Gorfine, H. , Day, R. , Bardos, D. , Taylor, B. , Prince, J. , Sainsbury, K. , & Dichmont, C. (2008). Rapid response to abalone virus depletion in Western Victoria: Information acquisition and reef code assessment. The University of Melbourne.

[mec16499-bib-0037] Grogan, L. F. , Cashins, S. D. , Skerratt, L. F. , Berger, L. , McFadden, M. S. , Harlow, P. , Hunter, D. A. , Scheele, B. C. , & Mulvenna, J. (2018). Evolution of resistance to chytridiomycosis is associated with a robust early immune response. Molecular Ecology, 27, 919–934. 10.1111/mec.14493 29337419

[mec16499-bib-0038] Guarna, M. M. , Hoover, S. E. , Huxter, E. , Higo, H. , Moon, K.‐M. , Domanski, D. , Bixby, M. E. F. , Melathopoulos, A. P. , Ibrahim, A. , Peirson, M. , Desai, S. , Micholson, D. , White, R. , Borchers, C. H. , Currie, R. W. , Pernal, S. F. , & Foster, L. J. (2017). Peptide biomarkers used for the selective breeding of a complex polygenic trait in honey bees. Scientific Reports, 7, 8381. 10.1038/s41598-017-08464-2 28827652 PMC5566959

[mec16499-bib-0039] Günther, T. , & Coop, G. (2013). Robust identification of local adaptation from allele frequencies. Genetics, 195(1), 205–220. 23821598 10.1534/genetics.113.152462 PMC3761302

[mec16499-bib-0040] Gusev, A. , Lee, S. H. , Trynka, G. , Finucane, H. , Vilhjálmsson, B. J. , Xu, H. , Zang, C. , Ripke, S. , Bulik‐Sullivan, B. , Stahl, E. , & Neale, B. M. (2014). Partitioning heritability of regulatory and cell‐type‐specific variants across 11 common diseases. The American Journal of Human Genetics, 95, 535–552. 25439723 10.1016/j.ajhg.2014.10.004 PMC4225595

[mec16499-bib-0041] Gutierrez, A. P. , Matika, O. , Bean, T. P. , & Houston, R. D. (2018). Genomic selection for growth traits in Pacific oyster (*Crassostrea gigas*): Potential of low‐density marker panels for breeding value prediction. Frontiers in Genetics, 9, 391. 10.3389/fgene.2018.00391 30283494 PMC6156352

[mec16499-bib-0042] Gwack, Y. , Byun, H. , Hwang, S. , Lim, C. , & Choe, J. (2001). CREB‐binding protein and histone deacetylase regulate the transcriptional activity of Kaposi’s sarcoma‐associated herpesvirus open reading frame 50. Journal of Virology, 75, 1909–1917. 10.1128/JVI.75.4.1909-1917.2001 11160690 PMC115137

[mec16499-bib-0043] Hansen, E. M. , Parke, J. L. , & Sutton, W. (2005). Susceptibility of Oregon forest trees and shrubs to Phytophthora ramorum: A comparison of artificial inoculation and natural infection. Plant Disease, 89, 63–70. 30795286 10.1094/PD-89-0063

[mec16499-bib-0044] Harvell, C. D. , & Lamb, J. B. (2020). Disease outbreaks can threaten marine biodiversity. In D. C. Behringer , B. R. Silliman , & K. D. Lafferty (Eds.), Marine disease ecology (pp. 141–158). Oxford Scholarship.

[mec16499-bib-0045] Harvell, C. D. , Mitchell, C. E. , Ward, J. R. , Altizer, S. , Dobson, A. P. , Ostfeld, R. S. , & Samuel, M. D. (2002). Ecology ‐ Climate warming and disease risks for terrestrial and marine biota. Science, 296, 2158–2162. 10.1126/science.1063699 12077394

[mec16499-bib-0046] Harvell, D. , Jordán‐Dahlgren, E. , Merkel, S. , Rosenberg, E. , Raymundo, L. , Smith, G. , Weil, E. , & Willis, B. (2007). Coral disease, environmental drivers, and the balance between coral and microbial associates. Oceanography, 20, 172–195. 10.5670/oceanog.2007.91

[mec16499-bib-0047] Hawley, D. M. , Osnas, E. E. , Dobson, A. P. , Hochachka, W. M. , Ley, D. H. , & Dhondt, A. A. (2013). Parallel patterns of increased virulence in a recently emerged wildlife pathogen. Plos Biology, 11(5), e1001570. 10.1371/journal.pbio.1001570 23723736 PMC3665845

[mec16499-bib-0048] Hivert, V. , Leblois, R. , Petit, E. J. , Gautier, M. , & Vitalis, R. (2018). Measuring genetic differentiation from pool‐seq data. Genetics, 210, 315–330. 10.1534/genetics.118.300900 30061425 PMC6116966

[mec16499-bib-0049] Hoffmann, A. A. , Miller, A. D. , & Weeks, A. R. (2020). Genetic mixing for population management: From genetic rescue to provenancing. Evolutionary Applications, 14, 634–652. 33767740 10.1111/eva.13154 PMC7980264

[mec16499-bib-0050] Hohenlohe, P. A. , Bassham, S. , Currey, M. , & Cresko, W. A. (2012). Extensive linkage disequilibrium and parallel adaptive divergence across threespine stickleback genomes. Philosophical Transactions of the Royal Society B, 367, 395–408. 10.1098/rstb.2011.0245 PMC3233713 22201169

[mec16499-bib-0051] Hohenlohe, P. A. , McCallum, H. I. , Jones, M. E. , Lawrance, M. F. , Hamede, R. K. , & Storfer, A. (2019). Conserving adaptive potential: Lessons from Tasmanian devils and their transmissible cancer. Conservation Genetics, 20, 81–87. 10.1007/s10592-019-01157-5 31551664 PMC6759055

[mec16499-bib-0052] Holland, O. J. , Young, M. A. , Sherman, C. D. H. , Tan, M. H. , Gorfine, H. , Matthews, T. Y. , & Miller, A. D. (2021). Ocean warming threatens key trophic interactions supporting a commercial fishery in a climate change hotspot. Global Change Biology, 27(24), 6498–6511. 34529873 10.1111/gcb.15889

[mec16499-bib-0053] Hooper, C. , Hardy‐Smith, P. , & Handlinger, J. (2007). Ganglioneuritis causing high mortalities in farmed Australian abalone (*Haliotis laevigata* and *Haliotis rubra*). Australian Veterinary Journal, 85, 188–193. 10.1111/j.1751-0813.2007.00155.x 17470067

[mec16499-bib-0054] Huang, D. W. , Sherman, B. T. , & Lempicki, R. A. (2009a). Bioinformatics enrichment tools: Paths toward the comprehensive functional analysis of large gene lists. Nucleic Acids Research, 37, 1–13. 10.1093/nar/gkn923 19033363 PMC2615629

[mec16499-bib-0055] Huang, D. W. , Sherman, B. T. , & Lempicki, R. A. (2009b). Systematic and integrative analysis of large gene lists using DAVID bioinformatics resources. Nucleic Acids Research, 37, 1–13. 19131956 10.1038/nprot.2008.211

[mec16499-bib-0056] Hubert, J. N. , Zerjal, T. , & Hospital, F. (2018). Cancer‐ and behavior‐related genes are targeted by selection in the Tasmanian devil (*Sarcophilus harrish*). PLoS One, 13, e0201838. 30102725 10.1371/journal.pone.0201838 PMC6089428

[mec16499-bib-0103] Jahanban‐Esfahlan, R. , Seidi, K. , Majidinia, M. , Karimian, A. , Yousefi, B. , Nabavi, S. M. , Astani, A. , Berindan‐Neagoe, I. , Gulei, D. , Fallarino, F. , Gargaro, M. , Manni, G. , Pirro, M. , Xu, S. , Sadeghi, M. , Nabavi, S. F. , & Shirooie, S. (2019). Toll‐like receptors as novel therapeutic targets for herpes simplex virus infection. Reviews in Medical Virology, 29(4), e2048. 10.1002/rmv.2048 31265195

[mec16499-bib-0057] Jeffreys, H. (1961). Theory of probability. Oxford University Press.

[mec16499-bib-0058] Johnson, C. R. , Banks, S. C. , Barrett, N. S. , Cazassus, F. , Dunstan, P. K. , Edgar, G. J. , Frusher, S. D. , Gardner, C. , Haddon, M. , Helidoniotis, F. , Hill, K. L. , Holbrook, N. J. , Hosie, G. W. , Last, P. R. , Ling, S. D. , Melbourne‐Thomas, J. , Miller, K. , Pecl, G. T. , Richardson, A. J. , … Taw, N. (2011). Climate change cascades: Shifts in oceanography, species’ ranges and subtidal marine community dynamics in eastern Tasmania. Journal of Experimental Marine Biology and Ecology, 400, 17–32. 10.1016/j.jembe.2011.02.032

[mec16499-bib-0059] Jombart, T. (2008). adegenet: a R package for the multivariate analysis of genetic markers. Bioinformatics, 24, 1403–1405. 10.1093/bioinformatics/btn129 18397895

[mec16499-bib-0060] Jombart, T. , & Ahmed, I. (2011). adegenet 1.3‐1: new tools for the analysis of genome‐wide SNP data. Bioinformatics, 27, 3070–3071. 10.1093/bioinformatics/btr521 21926124 PMC3198581

[mec16499-bib-0061] Kassambara, A. (2020). R package “Ggpubr”. Retrieved from https://cran.Rproject.org/web/packages/ggpubr/ggpubr.pdf

[mec16499-bib-0062] Kim, K. , & Harvell, C. D. (2004). The rise and fall of a six‐year coral‐fungal epizootic. American Naturalist, 164, S52–S63. 10.1086/424609 15540141

[mec16499-bib-0063] Kjøglum, S. , Henryon, M. , Aasmundstad, T. , & Korsgaard, I. (2008). Selective breeding can increase resistance of Atlantic salmon to furunculosis, infectious salmon anaemia and infectious pancreatic necrosis. Aquaculture, 39, 498–505. 10.1111/j.1365-2109.2008.01904.x

[mec16499-bib-0064] Lafferty, K. D. , Harvell, C. D. , Conrad, J. M. , Friedman, C. S. , Kent, M. L. , Kuris, A. M. , Powell, E. N. , Rondeau, D. , & Saksida, S. M. (2015). Infectious diseases affect marine fisheries and aquaculture economics. Annual Review of Marine Science, 7(7), 471–496. 10.1146/annurev-marine-010814-015646 25251276

[mec16499-bib-0065] Li, X. , Zhu, C. , Yeh, C.‐T. , Wu, W. , Takacs, E. M. , Petsch, K. A. , Tian, F. , Bai, G. , Buckler, E. S. , Muehlbauer, G. J. , Timmermans, M. C. P. , Scanlon, M. J. , Schnable, P. S. , & Yu, J. (2012). Genic and nongenic contributions to natural variation of quantitative traits in maize. Genome Research, 22, 2436–2444. 10.1101/gr.140277.112 22701078 PMC3514673

[mec16499-bib-0066] Ling, S. D. (2008). Range expansion of a habitat‐modifying species leads to loss of taxonomic diversity: a new and impoverished reef state. Oecologia, 156, 883–894. 10.1007/s00442-008-1043-9 18481099

[mec16499-bib-0067] Lorch, J. M. , Knowles, S. , Lankton, J. S. , Michell, K. , Edwards, J. L. , Kapfer, J. M. , Staffen, R. A. , Wild, E. R. , Schmidt, K. Z. , Ballmann, A. E. , Blodgett, D. , Farrell, T. M. , Glorioso, B. M. , Last, L. A. , Price, S. J. , Schuler, K. L. , Smith, C. E. , Wellehan, J. F. X. , & Blehert, D. S. (2016). Snake fungal disease: an emerging threat to wild snakes. Philosophical Transactions of the Royal Society B‐Biological Sciences, 371, 20150457. 10.1098/rstb.2015.0457 PMC5095536 28080983

[mec16499-bib-0068] Margres, M. J. , Jones, M. E. , Epstein, B. , Kerlin, D. H. , Comte, S. (2018). Large‐effect loci affect survival in Tasmanian devils (*Sarcophilus harrisii*) infected with a transmissible cancer. Molecular Ecology, 27, 4189–4199. 30171778 10.1111/mec.14853 PMC6759049

[mec16499-bib-0069] Martin, D. L. , Chiari, Y. , Boone, E. , Sherman, T. D. , Ross, C. , Wyllie‐Echeverria, S. , Gaydos, J. K. , & Boettcher, A. A. (2016). Functional, phylogenetic and host‐geographic signatures of *Labyrinthula* spp. Provide for putative species delimitation and a global‐scale view of seagrass wasting disease. Estuaries and Coasts, 39, 1403–1421. 10.1007/s12237-016-0087-z

[mec16499-bib-0070] Marty, G. D. , Hulson, P. , Miller, S. E. , TJ Quinn, I. I. , Moffitt, S. D. , & Merizon, R. A. (2010). Failure of population recovery in relation to disease in Pacific herring. Diseases of Aquatic Organisms, 90, 1–14. 10.3354/dao02210 20597425

[mec16499-bib-0071] Mayfield, S. , Mundy, C. , Gorfine, H. , Hart, A. , & Worthington, D. (2012). Fifty years of sustained production from the Australian abalone fisheries. Reviews in Fisheries Science, 20, 220–250. 10.1080/10641262.2012.725434

[mec16499-bib-0111] McCormick, C. , & Khaperskyy, D. A. (2017). Translation inhibition and stress granules in the antiviral immune response. Nature Reviews Immunology, 17(10), 647–660. 10.1038/nri.2017.63 28669985

[mec16499-bib-0072] Micheletti, S. J. , & Narum, S. R. (2018). Utility of pooled sequencing for association mapping in nonmodel organisms. Molecular Ecology Resources, 18, 825–837. 10.1111/1755-0998.12784 29633534

[mec16499-bib-0073] Miller, A. D. , Hoffmann, A. A. , Tan, M. H. , Young, M. , Ahrens, C. (2019) Local and regional scale habitat heterogeneity contribute to genetic adaptation in a commercially important marine mollusc (*Haliotis rubra*) from southeastern Australia. Molecular Ecology, 28, 3053–3072. 31077479 10.1111/mec.15128

[mec16499-bib-0074] Miller, A. D. , van Rooyen, A. , Rasic, G. , Ierodiaconou, D. A. , Gorfine, H. K. (2016). Contrasting patterns of population connectivity between regions in a commercially important mollusc Haliotis rubra: integrating population genetics, genomics and marine LiDAR data. Molecular Ecology, 25, 3845–3864. 27322873 10.1111/mec.13734

[mec16499-bib-0075] Montecino‐Latorre, D. , Eisenlord, M. E. , Turner, M. , Yoshioka, R. , Harvell, C. D. , Pattengill‐Semmens, C. V. , Nichols, J. D. , & Gaydos, J. K. (2016). Devastating transboundary impacts of sea star wasting disease on subtidal asteroids. PLoS One, 11, e0163190. 10.1371/journal.pone.0163190 27783620 PMC5082671

[mec16499-bib-0076] Moss, S. M. , Moss, D. R. , Arce, S. M. , Lightner, D. V. , & Lotz, J. M. (2012). The role of selective breeding and biosecurity in the prevention of disease in penaeid shrimp aquaculture. Journal of Invertebrate Pathology, 110, 247–250. 10.1016/j.jip.2012.01.013 22434005

[mec16499-bib-0078] Nam, B.‐H. , Jung, M. , Subramaniyam, S. , Yoo, S.‐I. , Markkandan, K. , Moon, J.‐Y. , Kim, Y.‐O. , Kim, D.‐G. , An, C. M. , Shin, Y. , Jung, H.‐J. , & Park, J.‐H. (2016). Transcriptome analysis revealed changes of multiple genes involved in Haliotis discus hannai innate immunity during Vibrio parahemolyticus Infection. PLoS One, 11, e0153474. 10.1371/journal.pone.0153474 27088873 PMC4835058

[mec16499-bib-0079] Neave, M. J. , Corbeil, S. , McColl, K. A. , & Crane, M. S. (2019). Investigating the natural resistance of blackfoot Paua Haliotis iris to abalone viral ganglioneuritis using whole transcriptome analysis. Diseases of Aquatic Organisms, 135, 107–119. 31342912 10.3354/dao03390

[mec16499-bib-0080] Oliver, E. C. J. , Benthuysen, J. A. , Bindoff, N. L. , Hobday, A. J. , Holbrook, N. J. , Mundy, C. N. , & Perkins‐Kirkpatrick, S. E. (2017). The unprecedented 2015/16 Tasman Sea marine heatwave. Nature Communications, 8, 16101. 10.1038/ncomms16101 PMC5519980 28706247

[mec16499-bib-0081] Potts, R. W. A. , Gutierrez, A. P. , Penaloza, C. S. , Regan, T. , Bean, T. P. , & Houston, R. D. (2021). Potential of genomic technologies to improve disease resistance in molluscan aquaculture. Philosophical Transactions of the Royal Society of London. Series B, Biological Sciences, 376, 20200168. 10.1098/rstb.2020.0168 33813884 PMC8059958

[mec16499-bib-0082] Qin, Y. , Jiang, S. , Huang, J. , Zhou, F. , Yang, Q. , Jiang, S. , & Yang, L. (2019). C‐type lectin response to bacterial infection and ammonia nitrogen stress in tiger shrimp (*Penaeus monodon*). Fish & Shellfish Immunology, 90, 188–198. 10.1016/j.fsi.2019.04.034 31028898

[mec16499-bib-0083] Ragone Calvo, L. M. , Calvo, G. W. , & Burreson, E. M. (2003). Dual disease resistance in a selectively bred eastern oyster, *Crassostrea virginica*, strain tested in Chesapeake Bay. Aquaculture, 220, 69–87. 10.1016/S0044-8486(02)00399-X

[mec16499-bib-0084] Schiebelhut, L. M. , Puritz, J. B. , & Dawson, M. N. (2018). Decimation by sea star wasting disease and rapid genetic change in a keystone species, *Pisaster ochraceus* . Proceedings of the National Academy of Sciences of the United States of America, 115, 7069–7074. 29915091 10.1073/pnas.1800285115 PMC6142263

[mec16499-bib-0106] Schmidt, T. , Striebinger, H. , Haas, J. , & Bailer, S. M. (2010). The heterogeneous nuclear ribonucleoprotein K is important for Herpes simplex virus‐1 propagation. FEBS Letters, 584(20), 4361–4365. 10.1016/j.febslet.2010.09.038 20888333

[mec16499-bib-0108] Shiel, B. P. , Hall, N. E. , Cooke, I. R. , Robinson, N. A. , & Strugnell, J. M. (2015). De novo characterisation of the greenlip abalone transcriptome (Haliotis laevigata) with a focus on the heat shock protein 70 (HSP70) family. Marine Biotechnology, 17(1), 23–32. 10.1007/s10126-014-9591-y 25079910

[mec16499-bib-0107] Silva, J. V. , Freitas, M. J. , Felgueiras, J. , & Fardilha, M. (2015). The power of the yeast two‐hybrid system in the identification of novel drug targets: Building and modulating PPP1 interactomes. Expert Review of Proteomics, 12(2), 147–158. 10.1586/14789450.2015.1024226 25795147

[mec16499-bib-0085] Skelly, D. A. , Magwene, P. M. , & Stone, E. A. (2016). Sporadic, global linkage disequilibrium between unlinked segregating sites. Genetics, 202, 427–437. 10.1534/genetics.115.177816 26715671 PMC4788226

[mec16499-bib-0101] Souki, S. K. , & Sandri‐Goldin, R. M. (2009). Arginine methylation of the ICP27 RGG box regulates the functional interactions of ICP27 with SRPK1 and Aly/REF during herpes simplex virus 1 infection. Journal of Virology, 83(17), 8970–8975. 10.1128/JVI.00801-09 19553338 PMC2738191

[mec16499-bib-0086] Storfer, A. , Kozakiewicz, C. P. , Beer, M. A. , & Savage, A. E. (2020). Applications of population genomics for understanding and mitigating wildlife disease. In P. A. Holenlohe , & O. P. Rajora (eds.), Population genomics: Wildlife, (pp. 357‐383). Springer.

[mec16499-bib-0098] Tang, S. , Patel, A. , & Krause, P. R. (2019). Hidden regulation of herpes simplex virus 1 pre‐mRNA splicing and polyadenylation by virally encoded immediate early gene ICP27. PLoS Path, 15(6), e1007884. 10.1371/journal.ppat.1007884 PMC6597130 31206552

[mec16499-bib-0099] Tang, X. , Huang, B. , Zhang, L. , Li, L. , & Zhang, G. (2017). Molecular characterization of Pacific oyster (Crassostrea gigas) IRAK4 gene and its role in MyD88‐dependent pathway. Developmental & Comparative Immunology, 72, 21–29. 10.1016/j.dci.2017.02.004 28223161

[mec16499-bib-0087] Tompkins, D. M. , Carver, S. , Jones, M. E. , Krkosek, M. , & Skerratt, L. F. (2015). Emerging infectious diseases of wildlife: A critical perspective. Trends in Parasitology, 31, 149–159. 10.1016/j.pt.2015.01.007 25709109

[mec16499-bib-0088] Uffelmann, E. , Huang, Q. Q. , Munung, N. S. , de Vries, J. , Okada, Y. , Martin, A. R. , Martin, H. C. , Lappalainen, T. , & Posthuma, D. (2021). Genome‐wide association studies. Nature Reviews Methods Primers, 1, 59. 10.1038/s43586-021-00056-9

[mec16499-bib-0089] Ujvari, B. , Pearse, A. M. , Swift, K. , Hodson, P. , Hua, B. (2014). Anthropogenic selection enhances cancer evolution in Tasmanian devil tumours. Evolutionary Applications, 7, 260–265. 24567746 10.1111/eva.12117 PMC3927887

[mec16499-bib-0102] Wang, X. W. , & Wang, J. X. (2013). Diversity and multiple functions of lectins in shrimp immunity. Developmental & Comparative Immunology, 39(1–2), 27–38. 10.1016/j.dci.2012.04.009 22561073

[mec16499-bib-0096] Wagner, L. M. , & DeLuca, N. A. (2013). Temporal association of herpes simplex virus ICP4 with cellular complexes functioning at multiple steps in PolII transcription. PLoS One, 8(10), e78242. 10.1371/journal.pone.0078242 24147125 PMC3795685

[mec16499-bib-0090] Wei, J. , Xie, W. , Li, R. , Wang, S. , Qu, H. , Ma, R. , Zhou, X. , & Jia, Z. (2020). Analysis of trait heritability in functionally partitioned rice genomes. Heredity, 124, 485–498. 10.1038/s41437-019-0244-9 31253955 PMC7029009

[mec16499-bib-0091] Weir, B. S. , & Cockerham, C. C. (1984). Estimating F‐statistics for the analysis of population structure. Evolution, 38, 1358–1370. 28563791 10.1111/j.1558-5646.1984.tb05657.x

[mec16499-bib-0092] Western Abalone Divers Association (2020). Assessment of abalone stocks in Western Zone Victoria: Submission to the TAC setting process for 2020. Port Fairy.

[mec16499-bib-0093] Willi, Y. , Kristensen, T. N. , Sgrò, C. M. , Weeks, A. R. , Ørsted, M. , & Hoffmann, A. A. (2022). Conservation genetics as a management tool: The five best‐supported paradigms to assist the management of threatened species. Proceedings of the National Academy of Sciences of the United States of America, 119, e2105076119. 10.1073/pnas.2105076119 34930821 PMC8740573

[mec16499-bib-0094] Young, M. A. , Treml, E. A. , Beher, J. , Fredle, M. , Gorfine, H. , Miller, A. D. , Swearer, S. E. , & Ierodiaconou, D. (2020). Using species distribution models to assess the long‐term impacts of changing oceanographic conditions on abalone density in south east Australia. Ecography, 43, 1052–1064. 10.1111/ecog.05181

[mec16499-bib-0095] Zhang, X.‐W. , Man, X. , Huang, X. , Wang, Y. , Song, Q.‐S. , Hui, K.‐M. , & Zhang, H.‐W. (2018). Identification of a C‐type lectin possessing both antibacterial and antiviral activities from red swamp crayfish. Fish & Shellfish Immunology, 77, 22–30. 10.1016/j.fsi.2018.03.015 29535012

[mec16499-bib-0196] Zhao, J. S. , Wang, A. Y. , Zhao, H. B. , & Chen, Y. H. (2017). Transcriptome sequencing and differential gene expression analysis of the schistosome‐transmitting snail *Oncomelania hupensis* inhabiting hilly and marshland regions. Scientific Reports, 7, 15809. 10.1038/s41598-017-16084-z 29150650 PMC5693929

