# Supplementary material for: Whole genome resequencing reveals signatures of rapid selection in a virus‐affected commercial fishery
Source: Mol Ecol. 2022 May 31;31(13):3658–71. doi: 10.1111/mec.16499 (PMC9327721; doi:10.1111/mec.16499)
Supplement: Supplementary file 1 — Fig S1‐2 [file MEC-31-3658-s001.docx]

**Whole genome resequencing reveals signatures of rapid selection in a virus affected commercial fishery**

Owen J. Holland, Madeline Toomey, Collin Ahrens, Ary A. Hoffmann, Laurence J. Croft, Craig D. H. Sherman, Adam D. Miller

**Supplemental Information**

**
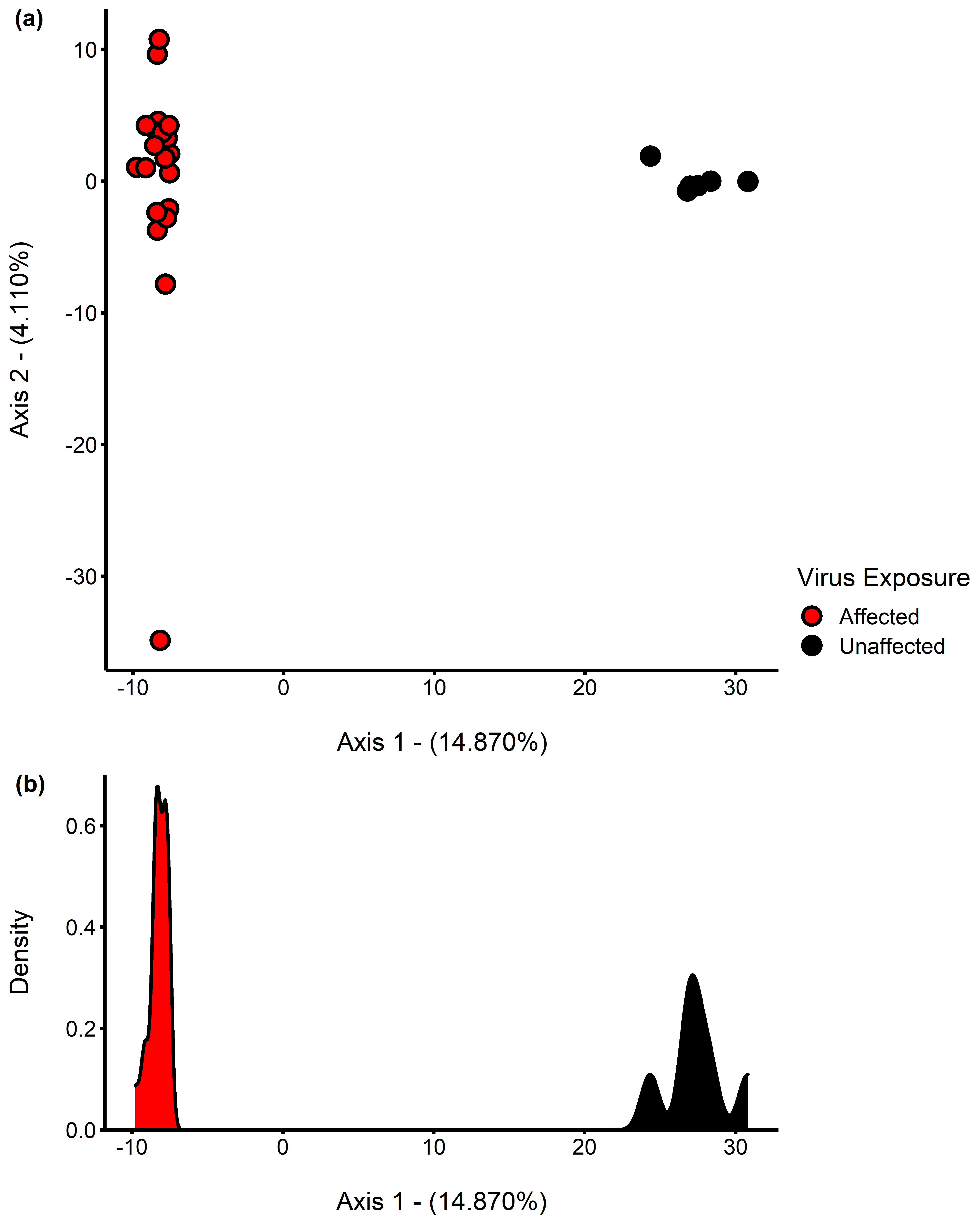
**

**Figure S1.** Plots of eigenvalues from the Principal Components Analysis, (a) plot of axes 1 and 2 eigenvalues, and (b) density plot of axis 1 eigen values. Plots are based on candidate SNP genotypes from 26 pooled whole genome resequencing libraries representing all virus affected fishing stocks (red) and all unaffected fishing stocks (black) except for site LJP.

**
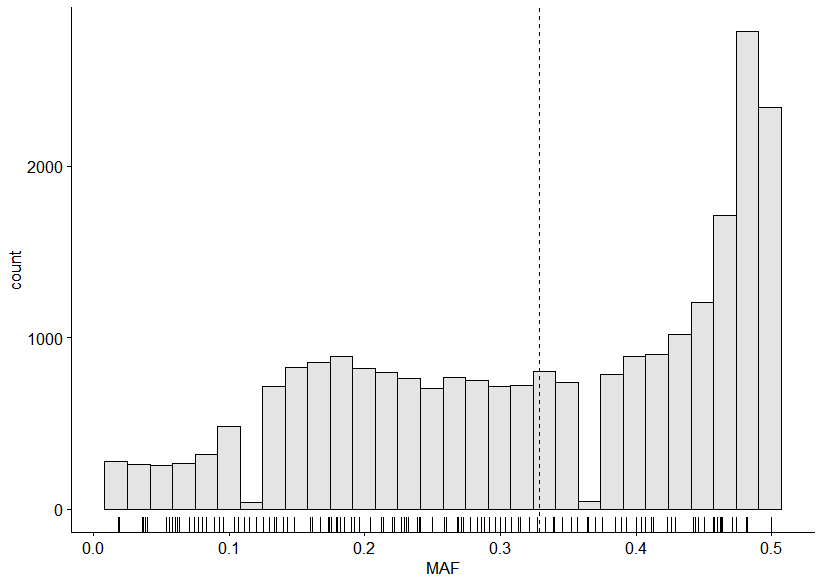
**

**Figure S2.** Histogram demonstrating the distribution of minor allele frequencies for all candidate SNP loci showing associations with virus exposure. The y-axis represents the total number of SNP loci and the x-axis represents minor allele frequency (MAF). The dashed vertical line indicates the mean frequency (0.32) for the minor alleles.
